# Supplementary material for: Vulnerable JavaScript functions detection using stacking of convolutional neural networks
Source: PeerJ Comput Sci. 2024 Feb 29;10:e1838. doi: 10.7717/peerj-cs.1838 (PMC10909211; doi:10.7717/peerj-cs.1838)
Supplement: Supplemental Information 2 [file peerj-cs-10-1838-s002.pdf]

Vulnerable JavaScript Functions Detection using Stacking of Convolutional Neural Networks (Supplementary Material)

Abdullah Sheneamer[1]

[1]**Department of Computer Science, Jazan University, Jazan, KSA**

Corresponding author:
Abdullah Sheneamer[1]

Email address: asheneamer@jazanu.edu.sa

ABSTRACT

Keywords:

DATA

- Ferenc et al's Datase Ferenc et al. (2019) `http://www.inf.u-szeged.hu/~ferenc/papers/JSVulnerabilityDataSet/`
- Viszkok et al's Dataset Viszkok et al. (2021) `https://security.snyk.io/`
- Apache Tomcat Dataset Ganesh et al. (2022) `https://github.com/palmafr/MDPIData2022/tree/main/datasets`
- Types of Vulnerabilities (Multi-Classes) Dataset Ganesh et al. (2021)

CODE

0.1 Machine learning for Binary classes

```


 # compare ensemble to each baseline classifier
 from numpy import mean
from numpy import std
from sklearn.datasets import make_classification
from sklearn.model_selection import cross_val_score
from sklearn.model_selection import RepeatedStratifiedKFold
from sklearn.linear_model import LogisticRegression
from sklearn.neighbors import KNeighborsClassifier
from sklearn.tree import DecisionTreeClassifier
from sklearn.svm import SVC
from sklearn.naive_bayes import GaussianNB
from sklearn.ensemble import StackingClassifier
from matplotlib import pyplot
import pandas as pd
from sklearn import svm
from sklearn.model_selection import GridSearchCV
import os
import matplotlib.pyplot as plt
#from skimage.transform import resize
import imread
import numpy as np
```

```
from sklearn.model_selection import train_test_split
from sklearn.metrics import classification_report,accuracy_score,
confusion_matrix
import pickle
# loading library
import numpy as np # linear algebra
import pandas as pd # data processing, CSV file I/O (e.g. pd.read_csv)
from sklearn.model_selection import train_test_split
from sklearn.neighbors import KNeighborsClassifier
from sklearn.ensemble import RandomForestClassifier
from sklearn.ensemble import BaggingClassifier
from sklearn.tree import DecisionTreeClassifier
from sklearn.naive_bayes import GaussianNB, BernoulliNB, MultinomialNB
from sklearn import svm
from imread import imread, imsave
from PIL import Image
 import numpy as np # linear algebra
import pandas as pd # data processing, CSV file I/O (e.g. pd.read_csv)
from sklearn.model_selection import train_test_split
from sklearn.preprocessing import MinMaxScaler
from keras.models import Model
from keras.layers import Input
import seaborn as sns
from keras.layers.core import Activation, Dropout, Dense
from sklearn import preprocessing
from sklearn.metrics import confusion_matrix
from sklearn.metrics import classification_report
import numpy as np # linear algebra
import pandas as pd # data processing, CSV file I/O (e.g. pd.read_csv)
from sklearn.model_selection import train_test_split
import sys
import os
from math import log
import scipy as sp
from imblearn.over_sampling import RandomOverSampler, SMOTE
from imblearn.under_sampling import RandomUnderSampler

import matplotlib.pyplot as plt
from keras.optimizers import SGD
import tensorflow as tf
from keras.models import Sequential
from keras.layers import Dropout, Dense, Conv1D, Flatten, MaxPooling1D
from sklearn.model_selection import train_test_split
from sklearn.datasets import load_iris
from numpy import unique
from keras.layers import Dense, Input, LSTM, Dropout, SimpleRNN, Embedding,
Reshape
from sklearn.metrics import confusion_matrix
from collections import Counter
from xgboost import XGBClassifier


df =  pd.read_csv('/Users/abdullah/Desktop/Folder/Research/
JSVulnerabilityDataSet-1.0.csv')
#df =  pd.read_csv('/Users/abdullah/Desktop/Folder/Research/
MDPIData2022-main/datasets/tomcat-final.csv')
#df =  pd.read_csv('/Users/abdullah/Desktop/Folder/Research/
MDPIData2022-main/datasets/struts-final.csv')



import matplotlib.pyplot as plt
df.columns
```

```
sns.countplot(x='Vuln', data=df)

X = df[[ 'CC', 'CCL', 'CCO', 'CI', 'CLC', 'CLLC', 'McCC',
        'NL', 'NLE', 'CD', 'CLOC', 'DLOC', 'TCD', 'TCLOC',
        'LLOC', 'LOC', 'NOS','NUMPAR', 'TLLOC', 'TLOC',
        'TNOS', 'HOR_D', 'HOR_T', 'HON_D', 'HON_T',
        'HLEN', 'HVOC', 'HDIFF', 'HVOL', 'HEFF',
        'HBUGS', 'HTIME', 'CYCL',  'PARAMS',
        'CYCL_DENS']].values



#y = df['Vuln']
y=df['Vuln']
# label_encoder object knows how to understand word labels.
label_encoder = preprocessing.LabelEncoder()

# Encode labels in column 'species'.
y = label_encoder.fit_transform(y)

counter = Counter(y)
print(counter)

#ros = RandomOverSampler(sampling_strategy={ 0: 10629,1: 10629},
random_state=42) # String
#X, y = ros.fit_resample(X, y)

#rus = RandomUnderSampler(sampling_strategy={ 0: 1496,1: 1496},
random_state=42) # String
#X, y = rus.fit_resample(X, y)

#smote = SMOTE() #SMOTE("minority")
#X, y= smote.fit_resample(X, y)



counter = Counter(y)
print(counter)


X_train, X_test, y_train, y_test = train_test_split(X,y,
test_size=0.33,shuffle=True, random_state=42, stratify=y)
X_test1=X_test
X_train1=X_train
y_test1=y_test
y_train1=y_train


from tensorflow.keras.utils import to_categorical
y_train = to_categorical(y_train)
y_test = to_categorical(y_test)



from sklearn.preprocessing import MinMaxScaler
scaler = MinMaxScaler()
scaler.fit(X_train)

Xtrain_scaled = scaler.transform(X_train)
Xtest_scaled = scaler.transform(X_test)

Xtrain_scaled.shape
```

```
#X_train, X_test, y_train, y_test=train_test_split(X,y, test_size=0.15,
random_state=42,stratify=y)
print('Splitted Successfully')


#␣get␣the␣dataset
#def␣get_dataset():
#␣␣␣␣␣␣␣X,␣y␣=␣make_classification(n_samples=1000,␣n_features=20,
n_informative=15,␣n_redundant=5,␣random_state=1)
#␣␣␣␣␣␣␣return␣X,␣y

#␣get␣a␣stacking␣ensemble␣of␣models
def␣get_stacking():
␣␣␣␣␣␣␣␣#␣define␣the␣base␣models
␣␣␣␣␣␣␣␣level0␣=␣list()
␣␣␣␣␣␣␣␣level0.append(('cart',␣DecisionTreeClassifier()))
␣␣␣␣␣␣␣␣level0.append(('lr',␣LogisticRegression(solver='liblinear',
␣␣␣␣max_iter=1000)))
␣␣␣␣␣␣␣␣level0.append(('bayes',␣GaussianNB()))
␣␣␣␣␣␣␣␣level0.append(('xgboost',␣XGBClassifier()))
␣␣␣␣␣␣␣␣level0.append(('bagging',␣BaggingClassifier()))
␣␣␣␣␣␣␣␣level0.append(('RF',␣RandomForestClassifier()))
␣␣␣␣␣␣␣␣level0.append(('knn',␣KNeighborsClassifier()))
␣␣␣␣␣␣␣␣level0.append(('svm',␣SVC()))

␣␣␣␣␣␣␣␣#␣define␣meta␣learner␣model
␣␣␣␣␣␣␣␣level1␣=␣LogisticRegression(solver='liblinear',max_iter=1000)
␣␣␣␣␣␣␣␣#␣define␣the␣stacking␣ensemble
␣␣␣␣␣␣␣␣model␣=␣StackingClassifier(estimators=level0,␣final_estimator=
␣level1,␣cv=10)
␣␣␣␣␣␣␣␣return␣model

#␣get␣a␣list␣of␣models␣to␣evaluate
def␣get_models():
␣␣␣␣␣␣␣␣models␣=␣dict()
␣␣␣␣␣␣␣␣models['cart']␣=␣DecisionTreeClassifier()
␣␣␣␣␣␣␣␣models['lr']␣=␣LogisticRegression(solver='liblinear',
␣max_iter=1000)
␣␣␣␣␣␣␣␣models['bayes']␣=␣GaussianNB()
␣␣␣␣␣␣␣␣models['xgboost']␣=␣XGBClassifier()
␣␣␣␣␣␣␣␣models['bagging']=BaggingClassifier()
␣␣␣␣␣␣␣␣models['rf']=RandomForestClassifier()
␣␣␣␣␣␣␣␣models['knn']␣=␣KNeighborsClassifier()
␣␣␣␣␣␣␣␣models['svm']␣=␣SVC()
␣␣␣␣␣␣␣␣models['stacking']␣=␣get_stacking()


␣␣␣␣␣␣␣␣return␣models

from␣sklearn.metrics␣import␣classification_report,␣accuracy_score,
make_scorer

#␣evaluate␣a␣give␣model␣using␣cross-validation
def␣evaluate_model(model,␣X,␣y,name):
␣␣␣␣␣␣␣␣cv␣=␣RepeatedStratifiedKFold(n_splits=10,␣n_repeats=3,
␣random_state=1)
␣␣␣␣␣␣␣␣print("====================================="+name+"=================
␣=====================================")
```

```
        #scores = cross_val_score(model, X, y,
    scoring=make_scorer(classification_report_with_accuracy_score)
 , cv=cv, n_jobs=-1, error_score='raise')
        scores = cross_val_score(model, X, y, scoring="f1", cv=cv,
 n_jobs=-1, error_score='raise')
        return scores


# get the models to evaluate
models = get_models()
# evaluate the models and store results
results, names = list(), list()
for name, model in models.items():
        #scores = evaluate_model(model, X, y,name)
        #results.append(scores)
        #names.append(name)
        print("==================================="+name+"=========
 ========================================")
        #print(name)
        cv = RepeatedStratifiedKFold(n_splits=10, n_repeats=3,
 random_state=42)
        #cvs1 = cross_val_score(model, X, y, scoring=
    make_scorer(classification_report_with_accuracy_score),
    cv=cv, n_jobs=-1, error_score='raise').mean()
        for score in ["roc_auc", "f1", "precision", "recall", "accuracy"]:
                cvs = cross_val_score(model, X, y, scoring=score, cv=cv,
        n_jobs=-1, error_score='raise').mean()
                print(score + " : "+ str(cvs))
        #print(cvs1)
        print('\n')
pyplot.boxplot(results, labels=names, showmeans=True)
pyplot.show()
```

0.2 Deep learning for Binary classes

```
 import numpy as np # linear algebra
import pandas as pd # data processing, CSV file I/O (e.g. pd.read_csv)
from sklearn.model_selection import train_test_split
from sklearn.preprocessing import MinMaxScaler
from keras.models import Model
from keras.layers import Input
import seaborn as sns
from keras.layers.core import Activation, Dropout, Dense
from sklearn import preprocessing
from sklearn.metrics import confusion_matrix
from sklearn.metrics import classification_report
import numpy as np # linear algebra
import pandas as pd # data processing, CSV file I/O (e.g. pd.read_csv)
from sklearn.model_selection import train_test_split
import sys
import os
from math import log
import scipy as sp
from imblearn.over_sampling import RandomOverSampler, SMOTE
from imblearn.under_sampling import RandomUnderSampler

import matplotlib.pyplot as plt
from keras.optimizers import SGD
import tensorflow as tf
from keras.models import Sequential
from keras.layers import Dropout, Dense, Conv1D, Flatten, MaxPooling1D
from sklearn.model_selection import train_test_split
```

```
from sklearn.datasets import load_iris
from numpy import unique
from keras.layers import Dense, Input, LSTM, Dropout, SimpleRNN,
Embedding, Reshape
from sklearn.metrics import confusion_matrix
from collections import Counter

df =  pd.read_csv('/Users/abdullah/Desktop/Folder/Research/
JSVulnerabilityDataSet-1.0.csv')
import matplotlib.pyplot as plt
sns.countplot(x='Vuln', data=df)

X = df[[ 'CC', 'CCL', 'CCO', 'CI', 'CLC', 'CLLC', 'McCC',
        'NL', 'NLE', 'CD', 'CLOC', 'DLOC', 'TCD', 'TCLOC',
        'LLOC', 'LOC', 'NOS','NUMPAR', 'TLLOC', 'TLOC',
        'TNOS', 'HOR_D', 'HOR_T', 'HON_D', 'HON_T','HLEN',
        'HVOC', 'HDIFF', 'HVOL', 'HEFF', 'HBUGS', 'HTIME',
        'CYCL', 'PARAMS', 'CYCL_DENS']].values

y = df['Vuln']

y

# label_encoder object knows how to understand word labels.
label_encoder = preprocessing.LabelEncoder()

# Encode labels in column 'species'.
y = label_encoder.fit_transform(y)

counter = Counter(y)
print(counter)

#ros = RandomOverSampler(sampling_strategy={ 0: 10629,1: 10629},
random_state=42) # String
#X, y = ros.fit_resample(X, y)

#rus = RandomUnderSampler(sampling_strategy={ 0: 1496,1: 1496},
random_state=42) # String
#X, y = rus.fit_resample(X, y)

#smote = SMOTE() #SMOTE("minority")
#X, y= smote.fit_resample(X, y)



counter = Counter(y)
print(counter)


X_train, X_test, y_train, y_test = train_test_split(X,y,
test_size=0.33,shuffle=True, random_state=42, stratify=y)
X_test1=X_test
X_train1=X_train
y_test1=y_test
y_train1=y_train


from tensorflow.keras.utils import to_categorical
y_train = to_categorical(y_train)
y_test = to_categorical(y_test)
```

```
from sklearn.preprocessing import MinMaxScaler
scaler = MinMaxScaler()
scaler.fit(X_train)

Xtrain_scaled = scaler.transform(X_train)
Xtest_scaled = scaler.transform(X_test)

Xtrain_scaled.shape


num_classes = 2

def model_VGG16(learning_rate=0.001, momentum=0.9):
    model = Sequential()


    model.add(Conv1D(64, 2, activation="relu", input_shape=(X.shape[1],1)))
    model.add(Conv1D(64, 2, activation='relu',
    kernel_initializer='he_uniform',padding='same'))
    model.add(MaxPooling1D())

    model.add(Conv1D(128,2, activation='relu',
    kernel_initializer='he_uniform',padding='same'))
    model.add(Conv1D(128, 2, activation='relu',
    kernel_initializer='he_uniform',
    padding='same'))
    model.add(MaxPooling1D())

    model.add(Conv1D(256, 2, activation='relu',
    kernel_initializer='he_uniform',padding='same'))
    model.add(Conv1D(256, 2, activation='relu',
    kernel_initializer='he_uniform',
    padding='same'))
    model.add(MaxPooling1D())

    model.add(Conv1D(512,2, activation='relu',
    kernel_initializer='he_uniform',padding='same'))
    model.add(Conv1D(512, 2, activation='relu',
    kernel_initializer='he_uniform',
    padding='same'))
    model.add(Conv1D(512, 2, activation='relu',
    kernel_initializer='he_uniform',
    padding='same'))
    model.add(MaxPooling1D())


    model.add(Conv1D(512,2, activation='relu',
    kernel_initializer='he_uniform',padding='same'))
    model.add(Conv1D(512, 2, activation='relu',
    kernel_initializer='he_uniform',
    padding='same'))
    model.add(Conv1D(512, 2, activation='relu',
    kernel_initializer='he_uniform',
    padding='same'))
    model.add(MaxPooling1D())


    model.add(Flatten())
    #malware_model.add(Dropout(0.5))

    model.add(Dense(4096, activation='relu',
```

```
    kernel_initializer='he_uniform'))
    model.add(Dropout(0.5))
    model.add(Dense(4096, activation='relu',
    kernel_initializer='he_uniform'))
    model.add(Dropout(0.5))
    model.add(Dense(1000, activation='relu',
    kernel_initializer='he_uniform'))

    model.add(Dense(num_classes, activation='sigmoid'))

    # compile model
    opt = SGD(lr=learning_rate, momentum=momentum)
    #model.compil(optimizer=opt,
    loss='categorical_crossentropy',
    metrics=[tf.keras.metrics.Precision(),
    tf.keras.metrics.Recall(), 'accuracy'])
    model.compile(loss='binary_crossentropy',
    optimizer=opt, metrics=['accuracy'])

    return model



def model_VGG19(learning_rate=0.001, momentum=0.9):


    model = Sequential()

    model.add(Conv1D(64, 2, activation="relu",
    input_shape=(X.shape[1],1)))
    model.add(Conv1D(64, 2, activation='relu',
    kernel_initializer='he_uniform',
    padding='same'))
    model.add(MaxPooling1D())

    model.add(Conv1D(128,2, activation='relu',
    kernel_initializer='he_uniform',padding='same'))
    model.add(Conv1D(128, 2, activation='relu',
    kernel_initializer='he_uniform', padding='same'))
    model.add(MaxPooling1D())

    model.add(Conv1D(256, 2, activation='relu',
    kernel_initializer='he_uniform', padding='same'))
    model.add(Conv1D(256, 2, activation='relu',
    kernel_initializer='he_uniform',
    padding='same'))
    model.add(MaxPooling1D())

    model.add(Conv1D(512,2, activation='relu',

    kernel_initializer='he_uniform',padding='same'))
    model.add(Conv1D(512, 2, activation='relu',
    kernel_initializer='he_uniform',padding='same'))
    model.add(Conv1D(512, 2, activation='relu',
    kernel_initializer='he_uniform',padding='same'))
    model.add(Conv1D(512, 2, activation='relu',
    kernel_initializer='he_uniform', padding='same'))
    model.add(MaxPooling1D())
```

```
    model.add(Conv1D(512,2, activation='relu',
    kernel_initializer='he_uniform',padding='same'))
    model.add(Conv1D(512, 2, activation='relu',
    kernel_initializer='he_uniform', padding='same'))
    model.add(Conv1D(512, 2, activation='relu',
    kernel_initializer='he_uniform', padding='same'))
    model.add(Conv1D(512, 2, activation='relu',
    kernel_initializer='he_uniform',padding='same'))
    model.add(MaxPooling1D())


    model.add(Flatten())
    #malware_model.add(Dropout(0.5))

    model.add(Dense(4096, activation='relu',
    kernel_initializer='he_uniform'))
    model.add(Dropout(0.5))
    model.add(Dense(4096, activation='relu',
    kernel_initializer='he_uniform'))
    model.add(Dropout(0.5))
    model.add(Dense(1000, activation='relu',
    kernel_initializer='he_uniform'))

    model.add(Dense(num_classes, activation='sigmoid'))

    # compile model
    opt = SGD(lr=learning_rate, momentum=momentum)
    #malware_model.compile(optimizer=opt,
    loss='categorical_crossentropy',
    metrics=[tf.keras.metrics.Precision(),
    tf.keras.metrics.Recall(), 'accuracy'])
    model.compile(loss='binary_crossentropy',
    optimizer=opt, metrics=['accuracy'])

    return model



def model_AlexNet(learning_rate=0.001, momentum=0.9):
    model = Sequential()

    model.add(Conv1D(96, 11, activation="relu",
    input_shape=(X.shape[1],1)))
    model.add(MaxPooling1D())

    model.add(Conv1D(256, 2, activation='relu',
    kernel_initializer='he_uniform',padding='same'))
    model.add(MaxPooling1D())

    model.add(Conv1D(384,2, activation='relu',
    kernel_initializer='he_uniform',padding='same'))
    model.add(Conv1D(384, 2, activation='relu',
    kernel_initializer='he_uniform',padding='same'))
    model.add(Conv1D(384, 2, activation='relu',
    kernel_initializer='he_uniform', padding='same'))
    model.add(MaxPooling1D())


    model.add(Flatten())
    model.add(Dense(4096, activation='relu',
```

```
    kernel_initializer='he_uniform'))
    model.add(Dropout(0.5))
    model.add(Dense(4096, activation='relu',
    kernel_initializer='he_uniform'))
    model.add(Dropout(0.5))
    model.add(Dense(1000, activation='relu',
    kernel_initializer='he_uniform'))
    model.add(Dense(num_classes, activation='sigmoid'))

    # compile model
    opt = SGD(lr=learning_rate, momentum=momentum)
    #malware_model.compile(optimizer=opt,
    loss='categorical_crossentropy',
    metrics=[tf.keras.metrics.Precision(),
    tf.keras.metrics.Recall(), 'accuracy'])
    model.compile(loss='binary_crossentropy',
    optimizer=opt, metrics=['accuracy'])
    return model


def model_Resent(learning_rate=0.001, momentum=0.9):
    model = Sequential()

    model.add(Conv1D(64, 2, activation="relu",
    input_shape=(X.shape[1],1)))
    model.add(Dropout(0.5))
    model.add(MaxPooling1D())

    model.add(Conv1D(64, 2, activation='relu',
    kernel_initializer='he_uniform',padding='same'))
    model.add(Conv1D(64, 2, activation='relu',
    kernel_initializer='he_uniform',padding='same'))
    model.add(Conv1D(64, 2, activation='relu',
    kernel_initializer='he_uniform',padding='same'))
    model.add(Conv1D(64, 2, activation='relu',
    kernel_initializer='he_uniform',padding='same'))

    model.add(Conv1D(128,2, activation='relu',
    kernel_initializer='he_uniform',padding='same'))
    model.add(Dropout(0.5))

    model.add(Conv1D(128, 2, activation='relu',
    kernel_initializer='he_uniform',padding='same'))
    model.add(Conv1D(128,2, activation='relu',
    kernel_initializer='he_uniform',padding='same'))
    model.add(Conv1D(128, 2, activation='relu',
    kernel_initializer='he_uniform',padding='same'))
    model.add(Conv1D(128,2, activation='relu',
    kernel_initializer='he_uniform',padding='same'))
    model.add(Conv1D(128, 2, activation='relu',
    kernel_initializer='he_uniform',padding='same'))
    model.add(Conv1D(128,2, activation='relu',
    kernel_initializer='he_uniform',padding='same'))
    model.add(Conv1D(128, 2, activation='relu',
    kernel_initializer='he_uniform',padding='same'))

    model.add(Conv1D(256, 2, activation='relu', kernel_initializer='he_uniform'
    padding='same'))
    model.add(Dropout(0.5))
```

```
model.add(Conv1D(256, 2, activation='relu',
kernel_initializer='he_uniform',padding='same'))
model.add(Conv1D(256, 2, activation='relu',
kernel_initializer='he_uniform',padding='same'))
model.add(Conv1D(256, 2, activation='relu',
kernel_initializer='he_uniform',padding='same'))
model.add(Conv1D(256, 2, activation='relu',
kernel_initializer='he_uniform',padding='same'))
model.add(Conv1D(256, 2, activation='relu',
kernel_initializer='he_uniform',padding='same'))
model.add(Conv1D(256, 2, activation='relu',
kernel_initializer='he_uniform',padding='same'))
model.add(Conv1D(256, 2, activation='relu',
kernel_initializer='he_uniform',padding='same'))
model.add(Conv1D(256, 2, activation='relu',
kernel_initializer='he_uniform',padding='same'))
model.add(Conv1D(256, 2, activation='relu',
kernel_initializer='he_uniform',padding='same'))
model.add(Conv1D(256, 2, activation='relu',
kernel_initializer='he_uniform',padding='same'))
model.add(Conv1D(256, 2, activation='relu',
kernel_initializer='he_uniform',padding='same'))

model.add(Conv1D(512,2, activation='relu',
kernel_initializer='he_uniform',padding='same'))
model.add(Dropout(0.5))

model.add(Conv1D(512, 2, activation='relu',
kernel_initializer='he_uniform',padding='same'))
model.add(Conv1D(512, 2, activation='relu',
kernel_initializer='he_uniform',padding='same'))
model.add(Conv1D(512, 2, activation='relu',
kernel_initializer='he_uniform',padding='same'))
model.add(Conv1D(512,2, activation='relu',
kernel_initializer='he_uniform',padding='same'))
model.add(Conv1D(512, 2, activation='relu',
kernel_initializer='he_uniform',padding='same'))

model.add(MaxPooling1D())


model.add(Flatten())
model.add(Dropout(0.5))

model.add(Dense(1000, activation='relu',
kernel_initializer='he_uniform'))

model.add(Dense(num_classes, activation='sigmoid'))

# compile model
opt = SGD(lr=learning_rate, momentum=momentum)
#malware_model.compile(optimizer=opt,
loss='categorical_crossentropy',
metrics=[tf.keras.metrics.Precision(),
tf.keras.metrics.Recall(), 'accuracy'])
model.compile(loss='binary_crossentropy',
optimizer=opt, metrics=['accuracy'])
```

```
    return model

def model_LSTM(learning_rate=0.001, momentum=0.9):

    input_layer = Input(shape=(X.shape[1],1))

    conv1 = Conv1D(filters=35,
                   kernel_size=8,
                   strides=1,
                   activation='relu')(input_layer)
    pool1 = MaxPooling1D(pool_size=4)(conv1)
    lstm1 = LSTM(35)(pool1)
    output_layer = Dense(2, activation='sigmoid')(lstm1)
    model = Model(inputs=input_layer, outputs=output_layer)
    opt = SGD(lr=learning_rate, momentum=momentum)
    model.compile(loss='binary_crossentropy',
    optimizer=opt, metrics=['acc'])

    return model



print(unique(y_test))
Vuln_model1 = model_VGG16()
Vuln_model2 = model_VGG19()
Vuln_model3 = model_AlexNet()
Vuln_model4 = model_Resent()
Vuln_model5 = model_LSTM()




y_train_new = np.argmax(y_train, axis=1)

y_train_new

from sklearn.utils import class_weight
from sklearn.utils import compute_class_weight

class_weights = compute_class_weight(
                                        class_weight = "balanced",
                                        classes = np.unique(y_train_new),
                                        y = y_train_new
                                    )
class_weights = dict(zip(np.unique(y_train_new), class_weights))
class_weights




history1 = Vuln_model1.fit(x= Xtrain_scaled,
y=y_train, batch_size=128, epochs=1000, verbose=1,
validation_split=0.33,class_weight=class_weights)
scores1 = Vuln_model1.evaluate(x= Xtest_scaled,
y=y_test, verbose=1)
print('Final Vuln_model1 (VGG16) accuracy: ', scores1[1])

plt.plot(history1.history['accuracy'])
plt.plot(history1.history['val_accuracy'])

plt.title('VGG16 model accuracy')
plt.ylabel('accuracy')
```

```
plt.xlabel('epoch')
plt.legend(['train','test'], loc='upper left')
plt.show()

plt.plot(history1.history['loss'])
plt.plot(history1.history['val_loss'])

plt.title('VGG16 model loss')
plt.ylabel('loss')
plt.xlabel('epoch')
plt.legend(['train','test'], loc='upper left')
plt.show()


classes = Vuln_model1.predict(x= Xtest_scaled)
y_classes = classes.argmax(axis=-1)
true_classes = y_test.argmax(axis=-1)

print(classification_report(true_classes, y_classes,digits=6))

cf_matrix = confusion_matrix(true_classes, y_classes)
print(cf_matrix)



history2 = Vuln_model2.fit(x= Xtrain_scaled,
y=y_train, batch_size=128, epochs=1000, verbose=1,
validation_split=0.33,class_weight=class_weights)
scores2 = Vuln_model2.evaluate(x= Xtest_scaled,
y=y_test, verbose=1)
print('Final Vuln_model2 (VGG19) accuracy: ', scores1[1])

import matplotlib.pyplot as plt2


print("Test Score:", scores2[0])
print("Test Accuracy:", scores2[1])

plt2.plot(history2.history['accuracy'])
plt2.plot(history2.history['val_accuracy'])

plt2.title('VGG19 model accuracy')
plt2.ylabel('accuracy')
plt2.xlabel('epoch')
plt2.legend(['train','test'], loc='upper left')
plt2.show()

plt2.plot(history2.history['loss'])
plt2.plot(history2.history['val_loss'])

plt2.title('VGG19 model loss')
plt2.ylabel('loss')
plt2.xlabel('epoch')
plt2.legend(['train','test'], loc='upper left')
plt2.show()


classes = Vuln_model2.predict(x= Xtest_scaled)
y_classes = classes.argmax(axis=-1)
true_classes = y_test.argmax(axis=-1)
```

```
print(classification_report(true_classes, y_classes,digits=6))


cf_matrix = confusion_matrix(true_classes, y_classes)
print(cf_matrix)
'''
group_names1 = ['True Neg','False Pos','False Neg','True Pos']
group_counts1 = ["{0:0.0f}".format(value) for value in
                 cf_matrix.flatten()]
group_percentages1 = ["{0:.2%}".format(value) for value in
                      cf_matrix.flatten()/np.sum(cf_matrix)]
labels1 = [f"{v1}\n{v2}\n{v3}" for v1, v2, v3 in
           zip(group_names1,group_counts1,group_percentages1)]
labels1 = np.asarray(labels1).reshape(2,2)
sns.heatmap(cf_matrix, annot=labels1, fmt='', cmap='Blues')
'''



import matplotlib.pyplot as plt3


history3 = Vuln_model3.fit(x= Xtrain_scaled,
y=y_train, batch_size=128, epochs=1000, verbose=1,
validation_split=0.33,class_weight=class_weights)
scores3 = Vuln_model3.evaluate(x= Xtest_scaled,
y=y_test, verbose=1)
print('Final Vuln_model3 (AlexNet) accuracy: ', scores3[1])


print("Test Score:", scores3[0])
print("Test Accuracy:", scores3[1])

plt3.plot(history3.history['accuracy'])
plt3.plot(history3.history['val_accuracy'])

plt3.title('AlexNet model accuracy')
plt3.ylabel('accuracy')
plt3.xlabel('epoch')
plt3.legend(['train','test'], loc='upper left')
plt3.show()

plt3.plot(history3.history['loss'])
plt3.plot(history3.history['val_loss'])

plt3.title('AlexNet model loss')
plt3.ylabel('loss')
plt3.xlabel('epoch')
plt3.legend(['train','test'], loc='upper left')
plt3.show()


classes = Vuln_model3.predict(x= Xtest_scaled)
y_classes = classes.argmax(axis=-1)
true_classes = y_test.argmax(axis=-1)
print(classification_report(true_classes, y_classes,digits=6))
```

```
cf_matrix = confusion_matrix(true_classes, y_classes)
print(cf_matrix)

import matplotlib.pyplot as plt4

history4 = Vuln_model4.fit(x= Xtrain_scaled,
y=y_train, batch_size=128, epochs=1000, verbose=1,
validation_split=0.33,class_weight=class_weights)
scores4 = Vuln_model4.evaluate(x= Xtest_scaled,
y=y_test, verbose=1)
print('Final␣Vuln_model4␣(Resent)␣accuracy:␣', scores4[1])


print("Test␣Score:", scores4[0])
print("Test␣Accuracy:", scores4[1])

plt4.plot(history4.history['accuracy'])
plt4.plot(history4.history['val_accuracy'])

plt4.title('Resent␣model␣accuracy')
plt4.ylabel('accuracy')
plt4.xlabel('epoch')
plt4.legend(['train','test'], loc='upper␣left')
plt4.show()

plt4.plot(history4.history['loss'])
plt4.plot(history4.history['val_loss'])

plt4.title('Resent␣model␣loss')
plt4.ylabel('loss')
plt4.xlabel('epoch')
plt4.legend(['train','test'], loc='upper␣left')
plt4.show()


classes = Vuln_model4.predict(x= Xtest_scaled)
y_classes = classes.argmax(axis=-1)
true_classes = y_test.argmax(axis=-1)
print(classification_report(true_classes, y_classes,digits=6))

cf_matrix = confusion_matrix(true_classes, y_classes)
print(cf_matrix)

history5 = Vuln_model5.fit(x= X_train, y=y_train,
batch_size=128, epochs=1000, verbose=1,
validation_split=0.33,class_weight=class_weights)
scores5 = Vuln_model5.evaluate(x= X_test,
y=y_test, verbose=1)
print('Final␣Vuln_model5␣(LSTM)␣accuracy:␣', scores5[1])

import matplotlib.pyplot as plt5
print("Test␣Score:", scores5[0])
print("Test␣Accuracy:", scores5[1])

plt5.plot(history5.history['acc'])
plt5.plot(history5.history['val_acc'])

plt5.title('LSTM␣model␣accuracy')
plt5.ylabel('accuracy')
plt5.xlabel('epoch')
```

```
plt5.legend(['train','test'], loc='upper left')
plt5.show()

plt5.plot(history5.history['loss'])
plt5.plot(history5.history['val_loss'])

plt5.title('LSTM model loss')
plt5.ylabel('loss')
plt5.xlabel('epoch')
plt5.legend(['train','test'], loc='upper left')
plt5.show()


classes = Vuln_model5.predict(x= X_test)
y_classes = classes.argmax(axis=-1)
true_classes = y_test.argmax(axis=-1)

print(classification_report(true_classes, y_classes,digits=6))



cf_matrix = confusion_matrix(true_classes, y_classes)
print(cf_matrix)

group_names = ['True Neg','False Pos','False Neg','True Pos']
group_counts = ["{0:0.0f}".format(value) for value in
                cf_matrix.flatten()]
group_percentages = ["{0:.2%}".format(value) for value in
                     cf_matrix.flatten()/np.sum(cf_matrix)]
labels = [f"{v1}\n{v2}\n{v3}" for v1, v2, v3 in
          zip(group_names,group_counts,group_percentages)]
labels = np.asarray(labels).reshape(2,2)
sns.heatmap(cf_matrix, annot=labels, fmt='', cmap='Blues')




Vuln_model1.save('/Users/abdullah/models/Model1.h5')
Vuln_model2.save('/Users/abdullah/models/Model2.h5')
Vuln_model3.save('/Users/abdullah/models/Model3.h5')
Vuln_model4.save('/Users/abdullah/models/Model4.h5')
Vuln_model5.save('/Users/abdullah/models/Model5.h5')



from keras.models import load_model

# load models from file
def load_all_models(n_models):
	all_models = list()
	for i in range(n_models):
		# define filename for this ensemble
		filename = '/Users/abdullah/models/Model' + str(i+1) + '.h5'
		# load model from file
		model = load_model(filename)
		# add to list of members
		all_models.append(model)
		print('>loaded \%s' \% filename)
	return all_models


```

```python
# load all models
n_members = 5
members = load_all_models(n_members)
print('Loaded \%d models' \% len(members))


 # create stacked model input dataset as outputs from the ensemble
def stacked_dataset(members, inputX):
        stackX = None
        for model in members:
                # make prediction
                yhat = model.predict(inputX, verbose=0)
                # stack predictions into [rows, members, probabilities]
                if stackX is None:
                        stackX = yhat
                else:
                        stackX = dstack((stackX, yhat))
        # flatten predictions to [rows, members x probabilities]
        stackX = stackX.reshape((stackX.shape[0],
              stackX.shape[1]*stackX.shape[2]))
        return stackX




 # fit a model based on the outputs from the ensemble members
def fit_stacked_model(members, inputX, inputy):
        # create dataset using ensemble
        stackedX = stacked_dataset(members, inputX)
        # fit standalone model
        model = LogisticRegression()
        model.fit(stackedX, inputy)
        return model


# fit stacked model using the ensemble
from numpy import dstack
from sklearn.linear_model import LogisticRegression
y_test=np.argmax(y_test, axis=1)

model = fit_stacked_model(members, Xtest_scaled, y_test)



# make a prediction with the stacked model

def stacked_prediction(members, model, inputX):
        # create dataset using ensemble
        stackedX = stacked_dataset(members, inputX)
        # make a prediction
        yhat = model.predict(stackedX)
        return yhat

# evaluate model on test set
from sklearn.metrics import accuracy_score
yhat = stacked_prediction(members, model, Xtest_scaled)

acc = accuracy_score(y_test, yhat)
print('Stacked Test Accuracy: \%.3f' \% acc)
print(len(yhat))


print(classification_report(y_test, yhat,digits=6))
```

```
cf_matrix = confusion_matrix(y_test, yhat)
print(cf_matrix)

group_names = ['True␣Neg','False␣Pos','False␣Neg','True␣Pos']
group_counts = ["{0:0.0f}".format(value) for value in
                cf_matrix.flatten()]
group_percentages = ["{0:.2\%}".format(value) for value in
                     cf_matrix.flatten()/np.sum(cf_matrix)]
labels = [f"{v1}\n{v2}\n{v3}" for v1, v2, v3 in
          zip(group_names,group_counts,group_percentages)]
labels = np.asarray(labels).reshape(2,2)
sns.heatmap(cf_matrix, annot=labels, fmt='', cmap='Blues')
}
```

0.3 Machine learning for Multi-classes

```
 # compare ensemble to each baseline classifier
from numpy import mean
from numpy import std
from sklearn.datasets import make_classification
from sklearn.model_selection import cross_val_score
from sklearn.model_selection import RepeatedStratifiedKFold
from sklearn.linear_model import LogisticRegression
from sklearn.neighbors import KNeighborsClassifier
from sklearn.tree import DecisionTreeClassifier
from sklearn.svm import SVC
from sklearn.naive_bayes import GaussianNB
from sklearn.ensemble import StackingClassifier
from matplotlib import pyplot
import pandas as pd
from sklearn import svm
from sklearn.model_selection import GridSearchCV
import os
import matplotlib.pyplot as plt
#from skimage.transform import resize
import imread
import numpy as np
from sklearn.model_selection import train_test_split
from sklearn.metrics import
classification_report,accuracy_score,confusion_matrix
import pickle
# loading library
import numpy as np # linear algebra
import pandas as pd # data processing, CSV file I/O (e.g. pd.read_csv)
from sklearn.model_selection import train_test_split
from sklearn.neighbors import KNeighborsClassifier
from sklearn.ensemble import RandomForestClassifier
from sklearn.ensemble import BaggingClassifier
from sklearn.tree import DecisionTreeClassifier
from sklearn.naive_bayes import GaussianNB, BernoulliNB, MultinomialNB
from sklearn import svm
from imread import imread, imsave
from PIL import Image
 import numpy as np # linear algebra
import pandas as pd # data processing, CSV file I/O (e.g. pd.read_csv)
from sklearn.model_selection import train_test_split
from sklearn.preprocessing import MinMaxScaler
from keras.models import Model
from keras.layers import Input
import seaborn as sns
from keras.layers.core import Activation, Dropout, Dense
from sklearn import preprocessing
from sklearn.metrics import confusion_matrix
```

```
from sklearn.metrics import classification_report
import numpy as np # linear algebra
import pandas as pd # data processing, CSV file I/O (e.g. pd.read_csv)
from sklearn.model_selection import train_test_split
import sys
import os
from math import log
import scipy as sp
from imblearn.over_sampling import RandomOverSampler, SMOTE
from imblearn.under_sampling import RandomUnderSampler

import matplotlib.pyplot as plt
from keras.optimizers import SGD
import tensorflow as tf
from keras.models import Sequential
from keras.layers import Dropout, Dense, Conv1D, Flatten, MaxPooling1D
from sklearn.model_selection import train_test_split
from sklearn.datasets import load_iris
from numpy import unique
from keras.layers import Dense, Input, LSTM, Dropout, SimpleRNN, Embedding, Res
from sklearn.metrics import confusion_matrix
from collections import Counter
from xgboost import XGBClassifier
import matplotlib.pyplot as plt

df =pd.read_csv('/Users/abdullah/Desktop/Folder/
Research/Vulnerability_Research/Vul_Datasets/
Code_Metrics_Datasets/Multi-Classes/PU_Dataset.csv')
df1 =pd.read_csv('/Users/abdullah/Desktop/Folder/
Research/Vulnerability_Research/Vul_Datasets/
Code_Metrics_Datasets/Multi-Classes/cwe119_GCDFile.csv')
df2 =pd.read_csv('/Users/abdullah/Desktop/Folder/Research/
Vulnerability_Research/Vul_Datasets/
Code_Metrics_Datasets/Multi-Classes/cwe399_cgd.csv')
df3 =pd.read_csv('/Users/abdullah/Desktop/Folder
/Research/Vulnerability_Research/Vul_Datasets/
Code_Metrics_Datasets/Multi-Classes/API_function_call.csv')
df4 =pd.read_csv('/Users/abdullah/Desktop/Folder
/Research/Vulnerability_Research/Vul_Datasets/
Code_Metrics_Datasets/Multi-Classes/Array_usage.csv')
df5 =pd.read_csv('/Users/abdullah/Desktop/Folder/
Research/Vulnerability_Research/Vul_Datasets/
Code_Metrics_Datasets/Multi-Classes/Arithmetic_expression.csv')

import matplotlib.pyplot as plt
df.columns


sns.countplot(x='IsVulnerable', data=df)

X = df[['Lines_Of_Program','Physic_Lines',
        'n1_Number_Of_Distinct_Operators',
        'n2_Number_Of_Distinct_Operands','n_Program_Vocabulary',
        'N1_Total_Number_Of_Operators','N2_Total_Number_Of_Operands',
        'N_Program_Length','B_Number_of_Delivered_Bugs_1',
        'B_Number_of_Delivered_Bugs_2',
        'D_Difficulty','E_Effort','T_Time_Required_To_Program',
        'V_Volume','_N_Calculated_Program_Length',
        'McCab_Number']].values


y= df['IsVulnerable'].values
```

```
print(len(y))



import matplotlib.pyplot as plt1
df1.columns


sns.countplot(x='IsVulnerable', data=df1)

X1 = df1[['Lines_Of_Program','Physic_Lines',
          'n1_Number_Of_Distinct_Operators',
          'n2_Number_Of_Distinct_Operands','n_Program_Vocabulary',
          'N1_Total_Number_Of_Operators','N2_Total_Number_Of_Operands',
          'N_Program_Length','B_Number_of_Delivered_Bugs_1',
          'B_Number_of_Delivered_Bugs_2','D_Difficulty','E_Effort',
          'T_Time_Required_To_Program','V_Volume',
          '_N_Calculated_Program_Length',
          'McCab_Number']].values

y1= df1['IsVulnerable'].values


print(len(y1))


X2 = df2[['Lines_Of_Program','Physic_Lines',
          'n1_Number_Of_Distinct_Operators',
          'n2_Number_Of_Distinct_Operands','n_Program_Vocabulary',
          'N1_Total_Number_Of_Operators','N2_Total_Number_Of_Operands',
          'N_Program_Length','B_Number_of_Delivered_Bugs_1',
          'B_Number_of_Delivered_Bugs_2','D_Difficulty',
          'E_Effort','T_Time_Required_To_Program','V_Volume',
          '_N_Calculated_Program_Length',
          'McCab_Number']].values


y2= df2['IsVulnerable'].values






print(len(y2))



X3 = df3[['Lines_Of_Program','Physic_Lines',
          'n1_Number_Of_Distinct_Operators','n2_Number_Of_Distinct_Operands',
          'n_Program_Vocabulary','N1_Total_Number_Of_Operators',
          'N2_Total_Number_Of_Operands','N_Program_Length',
          'B_Number_of_Delivered_Bugs_1','B_Number_of_Delivered_Bugs_2',
          'D_Difficulty','E_Effort','T_Time_Required_To_Program','V_Volume',
          '_N_Calculated_Program_Length','McCab_Number']].values

y3= df3['IsVulnerable'].values

print(len(y3))
```

```
X4 = df4[['Lines_Of_Program','Physic_Lines',
          'n1_Number_Of_Distinct_Operators',
          'n2_Number_Of_Distinct_Operands','n_Program_Vocabulary',
          'N1_Total_Number_Of_Operators','N2_Total_Number_Of_Operands',
          'N_Program_Length','B_Number_of_Delivered_Bugs_1',
          'B_Number_of_Delivered_Bugs_2','D_Difficulty',
          'E_Effort','T_Time_Required_To_Program','V_Volume',
          '_N_Calculated_Program_Length','McCab_Number']].values


y4= df4['IsVulnerable'].values

X5 = df5[['Lines_Of_Program','Physic_Lines',
        'n1_Number_Of_Distinct_Operators',
        'n2_Number_Of_Distinct_Operands',
        'n_Program_Vocabulary','N1_Total_Number_Of_Operators',
        'N2_Total_Number_Of_Operands','N_Program_Length',
        'B_Number_of_Delivered_Bugs_1','B_Number_of_Delivered_Bugs_2',
        'D_Difficulty','E_Effort','T_Time_Required_To_Program',
        'V_Volume','_N_Calculated_Program_Length',
        'McCab_Number']].values


y5= df5['IsVulnerable'].values


print(len(y5))


import numpy as np
X=np.concatenate((X,X1,X2,X3,X4,X5))
y=np.concatenate((y,y1,y2,y3,y4,y5))

import numpy
print(numpy.unique(y))

# label_encoder object knows how to understand word labels.
label_encoder = preprocessing.LabelEncoder()

# Encode labels in column 'species'.
y = label_encoder.fit_transform(y)
print(numpy.unique(y))

#from sklearn.preprocessing import label_binarize
#y = label_binarize(y, classes=[0, 1, 2, 3,4,5,6])

#counter = Counter(y)
#print(counter)

X_train, X_test, y_train, y_test = train_test_split(X,y,
test_size=0.33,shuffle=True, random_state=42, stratify=y)
X_test1=X_test
X_train1=X_train
y_test1=y_test
y_train1=y_train





from tensorflow.keras.utils import to_categorical
y_train = to_categorical(y_train)
y_test = to_categorical(y_test)
```

```
from sklearn.preprocessing import MinMaxScaler
scaler = MinMaxScaler()
scaler.fit(X_train)

Xtrain_scaled = scaler.transform(X_train)
Xtest_scaled = scaler.transform(X_test)

Xtrain_scaled.shape



#X_train, X_test, y_train, y_test=train_test_split(X, y, test_size=0.15,
random_state=42, stratify=y)
print('Splitted Successfully')


# get the dataset
#def get_dataset():
#        X, y = make_classification(n_samples=1000, n_features=20,
n_informative=15, n_redundant=5, random_state=1)
#        return X, y

# get a stacking ensemble of models
def get_stacking():
        # define the base models
        level0 = list()
        level0.append(('cart', DecisionTreeClassifier()))
        level0.append(('lr', LogisticRegression(solver='liblinear',
    max_iter=1000)))
        level0.append(('bayes', GaussianNB()))
        level0.append(('xgboost', XGBClassifier()))
        level0.append(('bagging', BaggingClassifier()))
        level0.append(('RF', RandomForestClassifier()))
        level0.append(('knn', KNeighborsClassifier()))
        level0.append(('svm', SVC()))

        # define meta learner model
        level1 = LogisticRegression(solver='liblinear',max_iter=1000)
        # define the stacking ensemble
        model = StackingClassifier(estimators=level0,
    final_estimator=level1, cv=10)
    return model

# get a list of models to evaluate
def get_models():
        models = dict()
        models['cart'] = DecisionTreeClassifier()
        models['lr'] = LogisticRegression(solver='liblinear',max_iter=1000)
        models['bayes'] = GaussianNB()
        models['xgboost'] = XGBClassifier()
        models['bagging']=BaggingClassifier()
        models['rf']=RandomForestClassifier()
        models['knn'] = KNeighborsClassifier()
        models['svm'] = SVC()
        models['stacking'] = get_stacking()


        return models
```

```
from sklearn.metrics import classification_report ,
accuracy_score , make_scorer

# evaluate a give model using cross-validation
def evaluate_model(model, X, y,name):
        cv = RepeatedStratifiedKFold(n_splits=10,
    n_repeats=3, random_state=1)
        print("=================================="+name+"====
     =============================================")
        #scores = cross_val_score(model, X, y, scoring=make_scorer(
              classification_report_with_accuracy_score),
                cv=cv, n_jobs=-1, error_score='raise')
        scores = cross_val_score(model, X, y, scoring="f1",
              cv=cv, n_jobs=-1, error_score='raise')
        return scores


# get the models to evaluate
models = get_models()
# evaluate the models and store results
results, names = list(), list()
for name, model in models.items():
        #scores = evaluate_model(model, X, y,name)
        #results.append(scores)
        #names.append(name)
        print("=================================="+name+"=========
           ==============================================")
        #print(name)
        cv = RepeatedStratifiedKFold(n_splits=10, n_repeats=3,
          random_state=42)
        for score in ["roc_auc", "f1", "precision",
                  "recall", "accuracy"]:
                cvs = cross_val_score(model, X, y,
                               scoring=score, cv=cv, n_jobs=-1,
                               error_score='raise').mean()
                print(score + " : "+ str(cvs))
        #print(cvs1)
        print('\n')
pyplot.boxplot(results, labels=names, showmeans=True)
pyplot.show()
```

0.4 Deep learning for Multi-classes

```
import numpy as np # linear algebra
import pandas as pd # data processing, CSV file I/O (e.g. pd.read_csv)
from sklearn.model_selection import train_test_split
from sklearn.preprocessing import MinMaxScaler
from keras.models import Model
from keras.layers import Input
import seaborn as sns
from keras.layers.core import Activation, Dropout, Dense
from sklearn import preprocessing
from sklearn.metrics import confusion_matrix
from sklearn.metrics import classification_report
import numpy as np # linear algebra
import pandas as pd # data processing, CSV file I/O (e.g. pd.read_csv)
from sklearn.model_selection import train_test_split
import sys
import os
from math import log
```

```
import scipy as sp
from imblearn.over_sampling import RandomOverSampler, SMOTE
from imblearn.under_sampling import RandomUnderSampler

import matplotlib.pyplot as plt
from keras.optimizers import SGD
import tensorflow as tf
from keras.models import Sequential
from keras.layers import Dropout, Dense, Conv1D, Flatten, MaxPooling1D
from sklearn.model_selection import train_test_split
from sklearn.datasets import load_iris
from numpy import unique
from keras.layers import Dense, Input, LSTM, Dropout, SimpleRNN,
Embedding, Reshape
from sklearn.metrics import confusion_matrix
from collections import Counter

df =  pd.read_csv('/Users/abdullah/Desktop/Folder/
Research/Vulnerability_Research/Vul_Datasets/Code_Metrics_Datasets/
Multi-Classes/PU_Dataset.csv')
df1 =  pd.read_csv('/Users/abdullah/Desktop/Folder/
Research/Vulnerability_Research/Vul_Datasets/Code_Metrics_Datasets/
Multi-Classes/cwe119_GCDFile.csv')
df2 =  pd.read_csv('/Users/abdullah/Desktop/Folder/
Research/Vulnerability_Research/Vul_Datasets/Code_Metrics_Datasets/
Multi-Classes/cwe399_cgd.csv')
df3 =  pd.read_csv('/Users/abdullah/Desktop/Folder/
Research/Vulnerability_Research/Vul_Datasets/Code_Metrics_Datasets/
Multi-Classes/API_function_call.csv')
df4 =  pd.read_csv('/Users/abdullah/Desktop/Folder/
Research/Vulnerability_Research/Vul_Datasets/Code_Metrics_Datasets/
Multi-Classes/Array_usage.csv')
df5 =  pd.read_csv('/Users/abdullah/Desktop/Folder/
Research/Vulnerability_Research/Vul_Datasets/Code_Metrics_Datasets/
Multi-Classes/Arithmetic_expression.csv')






import matplotlib.pyplot as plt
df.columns


sns.countplot(x='IsVulnerable', data=df)

X = df[['Lines_Of_Program','Physic_Lines',
          'n1_Number_Of_Distinct_Operators',
          'n2_Number_Of_Distinct_Operands','n_Program_Vocabulary',
          'N1_Total_Number_Of_Operators','N2_Total_Number_Of_Operands',
          'N_Program_Length','B_Number_of_Delivered_Bugs_1',
          'B_Number_of_Delivered_Bugs_2',
          'D_Difficulty','E_Effort','T_Time_Required_To_Program',
          'V_Volume','_N_Calculated_Program_Length',
          'McCab_Number']].values


y= df['IsVulnerable'].values



print(len(y))
```

```
import matplotlib.pyplot as plt1
df1.columns


sns.countplot(x='IsVulnerable', data=df1)

X1 = df1[['Lines_Of_Program','Physic_Lines',
          'n1_Number_Of_Distinct_Operators',
          'n2_Number_Of_Distinct_Operands','n_Program_Vocabulary',
          'N1_Total_Number_Of_Operators','N2_Total_Number_Of_Operands',
          'N_Program_Length','B_Number_of_Delivered_Bugs_1',
          'B_Number_of_Delivered_Bugs_2','D_Difficulty',
          'E_Effort','T_Time_Required_To_Program','V_Volume',
          '_N_Calculated_Program_Length','McCab_Number']].values


y1= df1['IsVulnerable'].values


print(len(y1))




X2 = df2[['Lines_Of_Program','Physic_Lines',
          'n1_Number_Of_Distinct_Operators',
          'n2_Number_Of_Distinct_Operands','n_Program_Vocabulary',
          'N1_Total_Number_Of_Operators','N2_Total_Number_Of_Operands',
          'N_Program_Length','B_Number_of_Delivered_Bugs_1',
          'B_Number_of_Delivered_Bugs_2', 'D_Difficulty','E_Effort',
          'T_Time_Required_To_Program','V_Volume',
          '_N_Calculated_Program_Length','McCab_Number']].values


y2= df2['IsVulnerable'].values






print(len(y2))



X3 = df3[['Lines_Of_Program','Physic_Lines',
          'n1_Number_Of_Distinct_Operators',
          'n2_Number_Of_Distinct_Operands','n_Program_Vocabulary',
          'N1_Total_Number_Of_Operators','N2_Total_Number_Of_Operands',
          'N_Program_Length','B_Number_of_Delivered_Bugs_1',
          'B_Number_of_Delivered_Bugs_2','D_Difficulty','E_Effort',
          'T_Time_Required_To_Program','V_Volume',
          '_N_Calculated_Program_Length','McCab_Number']].values



y3= df3['IsVulnerable'].values


print(len(y3))
```

```
X4 = df4[['Lines_Of_Program','Physic_Lines',
          'n1_Number_Of_Distinct_Operators',
          'n2_Number_Of_Distinct_Operands','n_Program_Vocabulary',
          'N1_Total_Number_Of_Operators','N2_Total_Number_Of_Operands',
          'N_Program_Length','B_Number_of_Delivered_Bugs_1',
          'B_Number_of_Delivered_Bugs_2',
          'D_Difficulty','E_Effort',
          'T_Time_Required_To_Program','V_Volume',
          '_N_Calculated_Program_Length','McCab_Number']].values


y4= df4['IsVulnerable'].values





X5 = df5[['Lines_Of_Program','Physic_Lines',
          'n1_Number_Of_Distinct_Operators',
          'n2_Number_Of_Distinct_Operands','n_Program_Vocabulary',
          'N1_Total_Number_Of_Operators',
          'N2_Total_Number_Of_Operands','N_Program_Length',
          'B_Number_of_Delivered_Bugs_1',
          'B_Number_of_Delivered_Bugs_2',
          'D_Difficulty','E_Effort',
          'T_Time_Required_To_Program','V_Volume',
          '_N_Calculated_Program_Length','McCab_Number']].values


y5= df5['IsVulnerable'].values


print(len(y5))


import numpy as np
X=np.concatenate((X,X1,X2,X3,X4,X5))
y=np.concatenate((y,y1,y2,y3,y4,y5))



# label_encoder object knows how to understand word labels.
label_encoder = preprocessing.LabelEncoder()

# Encode labels in column 'species'.
y = label_encoder.fit_transform(y)

counter = Counter(y)
print(counter)

#ros = RandomOverSampler(sampling_strategy={ 0: 85399,1: 85399},
random_state=42) # String
#X, y = ros.fit_resample(X, y)

#rus = RandomUnderSampler(sampling_strategy={ 0: 3475,1:
3475,2:3475,3:3475,4:3475,5:3475,6:3475}, random_state=42) # String
#X, y = rus.fit_resample(X, y)
```

```
#smote = SMOTE() #SMOTE("minority")
#X, y= smote.fit_resample(X, y)

rus = RandomUnderSampler(sampling_strategy={0:15000,1:
9952,3:13603,2:10440,4:7285,5:3475,6:10926}, random_state=42) # String
X, y = rus.fit_resample(X, y)



X_train, X_test, y_train, y_test = train_test_split(X,y,
test_size=0.33,shuffle=True, random_state=42, stratify=y)
X_test1=X_test
X_train1=X_train
y_test1=y_test
y_train1=y_train


from tensorflow.keras.utils import to_categorical
y_train = to_categorical(y_train)
y_test = to_categorical(y_test)



from sklearn.preprocessing import MinMaxScaler
scaler = MinMaxScaler()
scaler.fit(X_train)

Xtrain_scaled = scaler.transform(X_train)
Xtest_scaled = scaler.transform(X_test)

Xtrain_scaled.shape


num_classes = 7

def model_VGG16(learning_rate=0.001, momentum=0.9):
    model = Sequential()


    model.add(Conv1D(64, 2, activation="relu",
    input_shape=(X.shape[1],1)))
    model.add(Conv1D(64, 2, activation='relu',
    kernel_initializer='he_uniform',padding='same'))
    model.add(MaxPooling1D(pool_size=1))

    model.add(Conv1D(128,2, activation='relu',
    kernel_initializer='he_uniform',padding='same'))
    model.add(Conv1D(128, 2, activation='relu',
    kernel_initializer='he_uniform',padding='same'))
    model.add(MaxPooling1D(pool_size=1))

    model.add(Conv1D(256, 2, activation='relu',
    kernel_initializer='he_uniform',padding='same'))
    model.add(Conv1D(256, 2, activation='relu',
    kernel_initializer='he_uniform',padding='same'))
    model.add(MaxPooling1D(pool_size=1))

    model.add(Conv1D(512,2, activation='relu',
    kernel_initializer='he_uniform',padding='same'))
    model.add(Conv1D(512, 2, activation='relu',
    kernel_initializer='he_uniform',padding='same'))
```

```
    model.add(Conv1D(512, 2, activation='relu',
    kernel_initializer='he_uniform',padding='same'))
    model.add(MaxPooling1D(pool_size=1))


    model.add(Conv1D(512,2, activation='relu',
    kernel_initializer='he_uniform',padding='same'))
    model.add(Conv1D(512, 2, activation='relu',
    kernel_initializer='he_uniform',padding='same'))
    model.add(Conv1D(512, 2, activation='relu',
    kernel_initializer='he_uniform',padding='same'))
    model.add(MaxPooling1D(pool_size=1))


    model.add(Flatten())
    #malware_model.add(Dropout(0.5))

    model.add(Dense(4096, activation='relu',
    kernel_initializer='he_uniform'))
    model.add(Dropout(0.5))
    model.add(Dense(4096, activation='relu',
    kernel_initializer='he_uniform'))
    model.add(Dropout(0.5))
    model.add(Dense(1000, activation='relu',
    kernel_initializer='he_uniform'))

    model.add(Dense(num_classes, activation='softmax'))

    # compile model
    opt = SGD(lr=learning_rate, momentum=momentum)
    #malware_model.compile(optimizer=opt,
    loss='categorical_crossentropy',
    metrics=[tf.keras.metrics.Precision(),
    tf.keras.metrics.Recall(), 'accuracy'])
    model.compile(loss='categorical_crossentropy',
    optimizer=opt, metrics=['accuracy'])

    return model



def model_VGG19(learning_rate=0.01, momentum=0.9):


    model = Sequential()

    model.add(Conv1D(64, 2, activation="relu",
    input_shape=(X.shape[1],1)))
    model.add(Conv1D(64, 2, activation='relu',
    kernel_initializer='he_uniform',padding='same'))
    model.add(MaxPooling1D(pool_size=1))

    model.add(Conv1D(128,2, activation='relu',
    kernel_initializer='he_uniform',padding='same'))
    model.add(Conv1D(128, 2, activation='relu',
    kernel_initializer='he_uniform',padding='same'))
    model.add(MaxPooling1D(pool_size=1))

    model.add(Conv1D(256, 2, activation='relu',
    kernel_initializer='he_uniform',padding='same'))
```

```
    model.add(Conv1D(256, 2, activation='relu',
    kernel_initializer='he_uniform',padding='same'))
    model.add(MaxPooling1D(pool_size=1))

    model.add(Conv1D(512,2, activation='relu',
    kernel_initializer='he_uniform', padding='same'))
    model.add(Conv1D(512, 2, activation='relu',
    kernel_initializer='he_uniform',padding='same'))
    model.add(Conv1D(512, 2, activation='relu',
    kernel_initializer='he_uniform',padding='same'))
    model.add(Conv1D(512, 2, activation='relu',
    kernel_initializer='he_uniform',padding='same'))
    model.add(MaxPooling1D(pool_size=1))


    model.add(Conv1D(512,2, activation='relu',
    kernel_initializer='he_uniform',padding='same'))
    model.add(Conv1D(512, 2, activation='relu',
    kernel_initializer='he_uniform',padding='same'))
    model.add(Conv1D(512, 2, activation='relu',
    kernel_initializer='he_uniform',
    padding='same'))
    model.add(Conv1D(512, 2, activation='relu',
    kernel_initializer='he_uniform',padding='same'))
    model.add(MaxPooling1D(pool_size=1))


    model.add(Flatten())
    #malware_model.add(Dropout(0.5))

    model.add(Dense(4096, activation='relu',
    kernel_initializer='he_uniform'))
    model.add(Dropout(0.5))
    model.add(Dense(4096, activation='relu',
    kernel_initializer='he_uniform'))
    model.add(Dropout(0.5))
    model.add(Dense(1000, activation='relu',
    kernel_initializer='he_uniform'))

    model.add(Dense(num_classes, activation='softmax'))

    # compile model
    opt = SGD(lr=learning_rate, momentum=momentum)
    #malware_model.compile(optimizer=opt,
    loss='categorical_crossentropy',
    metrics=[tf.keras.metrics.Precision(),
    tf.keras.metrics.Recall(), 'accuracy'])
    model.compile(loss='categorical_crossentropy',
    optimizer=opt, metrics=['accuracy'])

    return model



def model_AlexNet(learning_rate=0.01, momentum=0.9):
    model = Sequential()

    model.add(Conv1D(96, 11, activation="relu",
    input_shape=(X.shape[1],1)))
    model.add(MaxPooling1D(pool_size=1))
```

```
    model.add(Conv1D(256, 2, activation='relu',
    kernel_initializer='he_uniform',padding='same'))
    model.add(MaxPooling1D(pool_size=1))

    model.add(Conv1D(384,2, activation='relu',
    kernel_initializer='he_uniform',padding='same'))
    model.add(Conv1D(384, 2, activation='relu',
    kernel_initializer='he_uniform',padding='same'))
    model.add(Conv1D(384, 2, activation='relu',
    kernel_initializer='he_uniform',padding='same'))
    model.add(MaxPooling1D(pool_size=1))


    model.add(Flatten())
    #malware_model.add(Dropout(0.5))

    model.add(Dense(4096, activation='relu',
    kernel_initializer='he_uniform'))
    model.add(Dropout(0.5))
    model.add(Dense(4096, activation='relu',
    kernel_initializer='he_uniform'))
    model.add(Dropout(0.5))
    model.add(Dense(1000, activation='relu',
    kernel_initializer='he_uniform'))

    model.add(Dense(num_classes, activation='softmax'))

    # compile model
    opt = SGD(lr=learning_rate, momentum=momentum)
    #malware_model.compile(optimizer=opt,
    loss='categorical_crossentropy',
    metrics=[tf.keras.metrics.Precision(),
    tf.keras.metrics.Recall(), 'accuracy'])
    model.compile(loss='categorical_crossentropy',
    optimizer=opt, metrics=['accuracy'])

    return model


def model_Resent(learning_rate=0.01, momentum=0.9):


    model = Sequential()

    model.add(Conv1D(64, 2, activation="relu",
    input_shape=(X.shape[1],1)))
    model.add(Dropout(0.5))
    model.add(MaxPooling1D(pool_size=1))

    model.add(Conv1D(64, 2, activation='relu',
    kernel_initializer='he_uniform',padding='same'))
    model.add(Conv1D(64, 2, activation='relu',
    kernel_initializer='he_uniform',padding='same'))
    model.add(Conv1D(64, 2, activation='relu',
    kernel_initializer='he_uniform',padding='same'))
    model.add(Conv1D(64, 2, activation='relu',
    kernel_initializer='he_uniform',padding='same'))

    model.add(Conv1D(128,2, activation='relu',
```

```
    kernel_initializer='he_uniform',padding='same'))
    model.add(Dropout(0.5))

    model.add(Conv1D(128, 2, activation='relu',
    kernel_initializer='he_uniform',padding='same'))
    model.add(Conv1D(128,2, activation='relu',
    kernel_initializer='he_uniform', padding='same'))
    model.add(Conv1D(128, 2, activation='relu',
    kernel_initializer='he_uniform',padding='same'))
    model.add(Conv1D(128,2, activation='relu',
    kernel_initializer='he_uniform',padding='same'))
    model.add(Conv1D(128, 2, activation='relu',
    kernel_initializer='he_uniform',padding='same'))
    model.add(Conv1D(128,2, activation='relu',
    kernel_initializer='he_uniform',padding='same'))
    model.add(Conv1D(128, 2, activation='relu',
    kernel_initializer='he_uniform',padding='same'))

    model.add(Conv1D(256, 2, activation='relu',
    kernel_initializer='he_uniform',padding='same'))
    model.add(Dropout(0.5))

    model.add(Conv1D(256, 2, activation='relu',
    kernel_initializer='he_uniform',padding='same'))
    model.add(Conv1D(256, 2, activation='relu',
    kernel_initializer='he_uniform',padding='same'))
    model.add(Conv1D(256, 2, activation='relu',
    kernel_initializer='he_uniform',padding='same'))
    model.add(Conv1D(256, 2, activation='relu',
    kernel_initializer='he_uniform',padding='same'))
    model.add(Conv1D(256, 2, activation='relu',
    kernel_initializer='he_uniform',padding='same'))
    model.add(Conv1D(256, 2, activation='relu',
    kernel_initializer='he_uniform',padding='same'))
    model.add(Conv1D(256, 2, activation='relu',
    kernel_initializer='he_uniform',padding='same'))
    model.add(Conv1D(256, 2, activation='relu',
    kernel_initializer='he_uniform',padding='same'))
    model.add(Conv1D(256, 2, activation='relu',
    kernel_initializer='he_uniform', padding='same'))
    model.add(Conv1D(256, 2, activation='relu',
    kernel_initializer='he_uniform',padding='same'))
    model.add(Conv1D(256, 2, activation='relu',
    kernel_initializer='he_uniform',padding='same'))

    model.add(Conv1D(512,2, activation='relu',
    kernel_initializer='he_uniform',padding='same'))
    model.add(Dropout(0.5))

    model.add(Conv1D(512, 2, activation='relu',
    kernel_initializer='he_uniform',padding='same'))
    model.add(Conv1D(512, 2, activation='relu',
    kernel_initializer='he_uniform',padding='same'))
    model.add(Conv1D(512, 2, activation='relu',
    kernel_initializer='he_uniform',padding='same'))
    model.add(Conv1D(512,2, activation='relu',
    kernel_initializer='he_uniform',padding='same'))
    model.add(Conv1D(512, 2, activation='relu',
```

```
    kernel_initializer='he_uniform',padding='same'))

    model.add(MaxPooling1D(pool_size=1))


    model.add(Flatten())
    model.add(Dropout(0.5))

    model.add(Dense(1000, activation='relu',
    kernel_initializer='he_uniform'))

    model.add(Dense(num_classes, activation='softmax'))

    # compile model
    opt = SGD(lr=learning_rate, momentum=momentum)
    #malware_model.compile(optimizer=opt,
    loss='categorical_crossentropy',
    metrics=[tf.keras.metrics.Precision(),
    tf.keras.metrics.Recall(), 'accuracy'])
    model.compile(loss='categorical_crossentropy',
    optimizer=opt, metrics=['accuracy'])

    return model

def model_LSTM(learning_rate=0.001, momentum=0.9):

    input_layer = Input(shape=(X.shape[1],1))

    conv1 = Conv1D(filters=35,
                kernel_size=8,
                strides=1,
                activation='relu')(input_layer)
    pool1 = MaxPooling1D(pool_size=4)(conv1)
    lstm1 = LSTM(35)(pool1)
    output_layer = Dense(7, activation='softmax')(lstm1)
    model = Model(inputs=input_layer, outputs=output_layer)
    opt = SGD(lr=learning_rate, momentum=momentum)
    model.compile(loss='categorical_crossentropy',
    optimizer=opt, metrics=['acc'])

    return model



print(unique(y_test))
Vuln_model1 = model_VGG16()
Vuln_model2 = model_VGG19()
Vuln_model3 = model_AlexNet()
Vuln_model4 = model_Resent()
Vuln_model5 = model_LSTM()




y_train_new = np.argmax(y_train, axis=1)

y_train_new

from sklearn.utils import class_weight
from sklearn.utils import compute_class_weight

class_weights =
```

```
compute_class_weight(

                        class_weight = "balanced",
                        classes = np.unique(y_train_new),
                        y = y_train_new
                    )
class_weights = dict(zip(np.unique(y_train_new), class_weights))
class_weights



history1 = Vuln_model1.fit(x= Xtrain_scaled,
y=y_train,batch_size=128, epochs=50, verbose=1,
validation_split=0.33,class_weight=class_weights)
scores1 = Vuln_model1.evaluate(x= Xtest_scaled,
y=y_test, verbose=1)
print('Final Vuln_model1 (VGG16) accuracy: ', scores1[1])



plt.plot(history1.history['accuracy'])
plt.plot(history1.history['val_accuracy'])

plt.title('VGG16 model accuracy')
plt.ylabel('accuracy')
plt.xlabel('epoch')
plt.legend(['train','test'], loc='upper left')
plt.show()

plt.plot(history1.history['loss'])
plt.plot(history1.history['val_loss'])

plt.title('VGG16 model loss')
plt.ylabel('loss')
plt.xlabel('epoch')
plt.legend(['train','test'], loc='upper left')
plt.show()


classes = Vuln_model1.predict(x= Xtest_scaled)
y_classes = classes.argmax(axis=-1)
true_classes = y_test.argmax(axis=-1)

print(classification_report(true_classes, y_classes,digits=6))

cf_matrix = confusion_matrix(true_classes, y_classes)
print(cf_matrix)

history2 = Vuln_model2.fit(x= Xtrain_scaled, y=y_train,
batch_size=128, epochs=20, verbose=1,
validation_split=0.33,class_weight=class_weights)
scores2 = Vuln_model2.evaluate(x= Xtest_scaled,
y=y_test, verbose=1)
print('Final Vuln_model2 (VGG19) accuracy: ', scores1[1])

import matplotlib.pyplot as plt2
```

```
plt2.plot(history2.history['accuracy'])
plt2.plot(history2.history['val_accuracy'])

plt2.title('VGG19 model accuracy')
plt2.ylabel('accuracy')
plt2.xlabel('epoch')
plt2.legend(['train','test'], loc='upper left')
plt2.show()

plt2.plot(history2.history['loss'])
plt2.plot(history2.history['val_loss'])

plt2.title('VGG19 model loss')
plt2.ylabel('loss')
plt2.xlabel('epoch')
plt2.legend(['train','test'], loc='upper left')
plt2.show()


classes = Vuln_model2.predict(x= Xtest_scaled)
y_classes = classes.argmax(axis=-1)
true_classes = y_test.argmax(axis=-1)

print(classification_report(true_classes, y_classes,digits=6))


cf_matrix = confusion_matrix(true_classes, y_classes)
print(cf_matrix)

import matplotlib.pyplot as plt3


history3 = Vuln_model3.fit(x= Xtrain_scaled, y=y_train,
batch_size=128, epochs=20, verbose=1,
validation_split=0.33,class_weight=class_weights)
scores3 = Vuln_model3.evaluate(x= Xtest_scaled,
y=y_test, verbose=1)
print('Final Vuln_model3 (AlexNet) accuracy: ', scores3[1])



plt3.plot(history3.history['accuracy'])
plt3.plot(history3.history['val_accuracy'])

plt3.title('AlexNet model accuracy')
plt3.ylabel('accuracy')
plt3.xlabel('epoch')
plt3.legend(['train','test'], loc='upper left')
plt3.show()

plt3.plot(history3.history['loss'])
plt3.plot(history3.history['val_loss'])

plt3.title('AlexNet model loss')
plt3.ylabel('loss')
plt3.xlabel('epoch')
plt3.legend(['train','test'], loc='upper left')
plt3.show()
```

```
classes = Vuln_model3.predict(x= Xtest_scaled)
y_classes = classes.argmax(axis=-1)
true_classes = y_test.argmax(axis=-1)
print(classification_report(true_classes, y_classes,digits=6))



cf_matrix = confusion_matrix(true_classes, y_classes)
print(cf_matrix)


import matplotlib.pyplot as plt4


history4 = Vuln_model4.fit(x= Xtrain_scaled,
y=y_train, batch_size=128, epochs=20, verbose=1,
validation_split=0.33,class_weight=class_weights)
scores4 = Vuln_model4.evaluate(x= Xtest_scaled,
y=y_test, verbose=1)
print('Final Vuln_model4 (Resent) accuracy: ', scores4[1])


plt4.plot(history4.history['accuracy'])
plt4.plot(history4.history['val_accuracy'])

plt4.title('Resent model accuracy')
plt4.ylabel('accuracy')
plt4.xlabel('epoch')
plt4.legend(['train','test'], loc='upper left')
plt4.show()

plt4.plot(history4.history['loss'])
plt4.plot(history4.history['val_loss'])

plt4.title('Resent model loss')
plt4.ylabel('loss')
plt4.xlabel('epoch')
plt4.legend(['train','test'], loc='upper left')
plt4.show()


classes = Vuln_model4.predict(x= Xtest_scaled)
y_classes = classes.argmax(axis=-1)
true_classes = y_test.argmax(axis=-1)
print(classification_report(true_classes, y_classes,digits=6))



cf_matrix = confusion_matrix(true_classes, y_classes)
print(cf_matrix)

history5 = Vuln_model5.fit(x= X_train, y=y_train,
batch_size=128, epochs=20, verbose=1,
validation_split=0.33,class_weight=class_weights)
scores5 = Vuln_model5.evaluate(x= X_test,
y=y_test, verbose=1)
print('Final Vuln_model5 (LSTM) accuracy: ', scores5[1])

import matplotlib.pyplot as plt5

plt5.plot(history5.history['acc'])
```

```
plt5.plot(history5.history['val_acc'])

plt5.title('LSTM model accuracy')
plt5.ylabel('accuracy')
plt5.xlabel('epoch')
plt5.legend(['train','test'], loc='upper left')
plt5.show()

plt5.plot(history5.history['loss'])
plt5.plot(history5.history['val_loss'])

plt5.title('LSTM model loss')
plt5.ylabel('loss')
plt5.xlabel('epoch')
plt5.legend(['train','test'], loc='upper left')
plt5.show()


classes = Vuln_model5.predict(x= X_test)
y_classes = classes.argmax(axis=-1)
true_classes = y_test.argmax(axis=-1)

print(classification_report(true_classes, y_classes,digits=6))



cf_matrix = confusion_matrix(true_classes, y_classes)
print(cf_matrix)

group_names = ['True Neg','False Pos','False Neg','True Pos']
group_counts = ["{0:0.0f}".format(value) for value in
                cf_matrix.flatten()]
group_percentages = ["{0:.2\%}".format(value) for value in
                     cf_matrix.flatten()/np.sum(cf_matrix)]
labels = [f"{v1}\n{v2}\n{v3}" for v1, v2, v3 in
          zip(group_names,group_counts,group_percentages)]
labels = np.asarray(labels).reshape(2,2)
sns.heatmap(cf_matrix, annot=labels, fmt='', cmap='Blues')




Vuln_model1.save('/Users/abdullah/models/Model1.h5')
Vuln_model2.save('/Users/abdullah/models/Model2.h5')
Vuln_model3.save('/Users/abdullah/models/Model3.h5')
Vuln_model4.save('/Users/abdullah/models/Model4.h5')
Vuln_model5.save('/Users/abdullah/models/Model5.h5')



from keras.models import load_model

# load models from file
def load_all_models(n_models):
        all_models = list()
        for i in range(n_models):
                # define filename for this ensemble
                filename = '/Users/abdullah/models/Model' + str(i+1) + '.h5'
                # load model from file
                model = load_model(filename)
                # add to list of members
```

```
			all_models.append(model)
			print('>loaded \%s' \% filename)
		return all_models


# load all models
n_members = 5
members = load_all_models(n_members)
print('Loaded \%d models' \% len(members))


 # create stacked model input dataset as outputs from the ensemble
def stacked_dataset(members, inputX):
		stackX = None
		for model in members:
			# make prediction
			yhat = model.predict(inputX, verbose=0)
			# stack predictions into [rows, members, probabilities]
			if stackX is None:
				stackX = yhat
			else:
				stackX = dstack((stackX, yhat))
		# flatten predictions to [rows, members x probabilities]
		stackX = stackX.reshape((stackX.shape[0],
 stackX.shape[1]*stackX.shape[2]))
		return stackX




 # fit a model based on the outputs from the ensemble members
def fit_stacked_model(members, inputX, inputy):
		# create dataset using ensemble
		stackedX = stacked_dataset(members, inputX)
		# fit standalone model
		model = LogisticRegression()
		model.fit(stackedX, inputy)
		return model


# fit stacked model using the ensemble
from numpy import dstack
from sklearn.linear_model import LogisticRegression
y_test=np.argmax(y_test, axis=1)

model = fit_stacked_model(members, Xtest_scaled, y_test)



# make a prediction with the stacked model

def stacked_prediction(members, model, inputX):
		# create dataset using ensemble
		stackedX = stacked_dataset(members, inputX)
		# make a prediction
		yhat = model.predict(stackedX)
		return yhat

# evaluate model on test set
from sklearn.metrics import accuracy_score
yhat = stacked_prediction(members, model, Xtest_scaled)

acc = accuracy_score(y_test, yhat)
```

```
print('Stacked Test Accuracy: \%.3f' \% acc)
print(len(yhat))



'''
y_classes = yhat.argmax(axis=-1)
true_classes = y_test.argmax(axis=-1)

print(yhat)
print(y_classes)
print(y_test)
print(true_classes)

for x in range(len(y_classes)):
    print(classes[x])
print("++++++")
print(true_classes)
for x in range(len(true_classes)):
    print(true_classes[x])
'''
print(classification_report(y_test, yhat, digits=6))

cf_matrix = confusion_matrix(y_test, yhat)
print(cf_matrix)

group_names = ['True Neg','False Pos','False Neg','True Pos']
group_counts = ["{0:0.0f}".format(value) for value in
                cf_matrix.flatten()]
group_percentages = ["{0:.2\%}".format(value) for value in
                     cf_matrix.flatten()/np.sum(cf_matrix)]
labels = [f"{v1}\n{v2}\n{v3}" for v1, v2, v3 in
          zip(group_names,group_counts,group_percentages)]
labels = np.asarray(labels).reshape(2,2)
sns.heatmap(cf_matrix, annot=labels, fmt='', cmap='Blues')
```

REFERENCES

Ferenc, R., Hegedűs, P., Gyimesi, P., Antal, G., Bán, D., and Gyimóthy, T. (2019). Challenging machine learning algorithms in predicting vulnerable javascript functions. In *2019 IEEE/ACM 7th International Workshop on Realizing Artificial Intelligence Synergies in Software Engineering (RAISE)*, pages 8–14. IEEE.

Ganesh, S., Ohlsson, T., and Palma, F. (2021). Predicting security vulnerabilities using source code metrics. In *2021 Swedish Workshop on Data Science (SweDS)*, pages 1–7. IEEE.

Ganesh, S., Palma, F., and Olsson, T. (2022). Are source code metrics “good enough” in predicting security vulnerabilities? *Data*, 7(9):127.

Viszkok, T., Hegedűs, P., and Ferenc, R. (2021). Improving vulnerability prediction of javascript functions using process metrics. *arXiv preprint arXiv:2105.07527*.
